# Pseudo-Hyperaldosteronism Arising from Licorice Cough Syrup Self-Ingestion: A Case Report

**DOI:** 10.3390/reports7040085

**Published:** 2024-10-14

**Authors:** Chien-Chun Liao, Kun-Te Lin

**Affiliations:** Department of Emergency and Critical Care Medicine, Changhua Christian Hospital, Changhua City 500209, Taiwan; 182929@cch.org.tw

**Keywords:** licorice, spironolactone, pseudo-hyperaldosteronism

## Abstract

**Background and Clinical Significance**: Licorice (glycyrrhiza glabra) cough syrup intoxication is manifested with refractory hypokalemia, hypertension, and metabolic alkalosis. The transformation of glycyrrhiza glabra metabolic into glycyrrhetic acid after ingestion further inhibits the 11-β-hydroxysteroid dehydrogenase-2 enzyme, impeding the conversion of cortisol into cortisone. The accumulation of cortisol can also stimulate mineralocorticoid receptors, which leads to a pseudo-hyperaldosteronism-like effect. **Case Presentation:** We report a 60-year-old male patient with licorice intoxication due to the chronic consumption of licorice cough syrup. He exhibited a transient seizure lasting approximately one minute. Initially, hypokalemia (potassium level was 2.0 mmol/L), metabolic alkalosis, and QT interval prolongation with premature ventricular complexes were demonstrated on his electrocardiogram. Despite the administration of both intravenous and oral potassium supplements over two days, there was no significant improvement in hypokalemia. Spironolactone, an aldosterone receptor antagonist, was administered in addition to ongoing potassium supplementation from the 3rd day. This intervention led to a rapid normalization of hypokalemia in one day. The patient was ultimately discharged on the 6th day without any subsequent complications. **Conclusions:** The licorice-induced chronic intoxication, which led to pseudo-hyperaldosteronism and refractory hypokalemia, was successfully managed with aggressive potassium supplementation and spironolactone treatment.

## 1. Introduction and Clinical Significance

The administration of licorice (glycyrrhiza glabra) cough syrup is common; however, the potential toxicity following prolonged exposure cannot be underestimated. Licorice undergoes a metabolic transformation into glycyrrhetic acid after ingestion. This compound inhibits the 11-β-hydroxysteroid dehydrogenase-2 enzyme, impeding the conversion of cortisol into cortisone [1]. The resultant accumulation of cortisol leads to the overstimulation of mineralocorticoid receptors, which can precipitate life-threatening conditions such as hypertension, hypokalemia, and metabolic alkalosis [2].

In a noteworthy case, we elucidate the complications arising from the chronic ingestion of licorice cough syrup, characterized by refractory hypokalemia, QT interval prolongation, and intermittent ventricular premature complexes.

## 2. Case Presentation

A 60-year-old male patient with controlled hypertension and schizophrenia, taking regular medication, presented with a two-month cough. He took citalopram (20 mg once daily), lurasidone (40 mg once daily), clonazepam (0.5 mg at bedtime), amlodipine/valsartan (5/80 mg once daily), and nebivolol (5 mg once daily) as part of his regular medication. In addition, he frequented a local medical facility every three days for cough medication and consumed various over-the-counter medications because he was worried about the Coronavirus Disease 2019 (COVID-19) infection. Notably, he ingested one bottle (120 mL) of licorice cough syrup daily as an antitussive for approximately one month.

**Figure 1:**
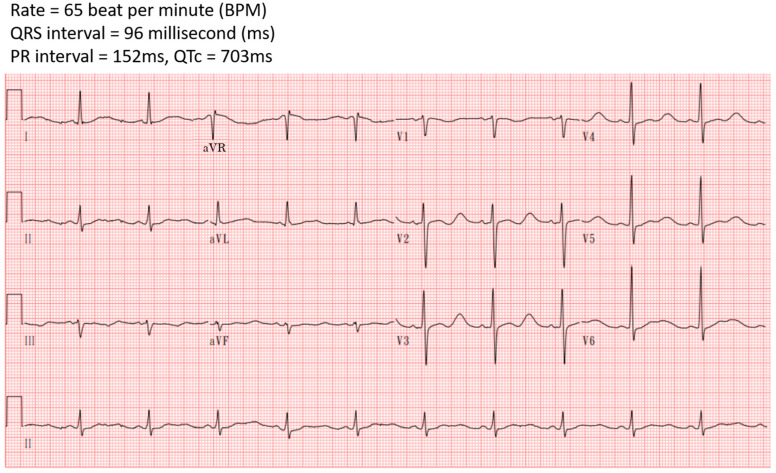
Initially electrocardiography in the emergency department showed QTc prolongation.

**Figure 2:**
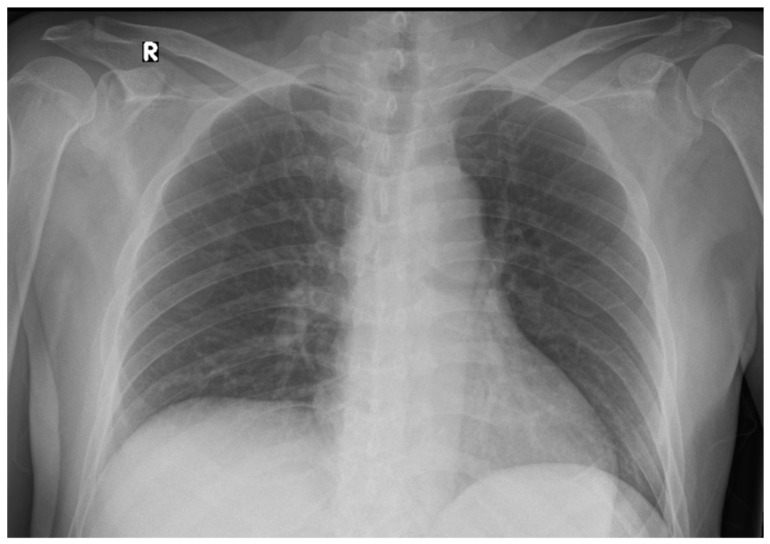
Chest X-ray in the emergency department showed unremarkable findings.

He experienced arm waving, an upward deviation in his eyes, shortness of breath, and cyanosis, which persisted for one minute. Subsequently, he was taken to the emergency department. His vital signs were stable, and he exhibited no neurological deficits. Blood tests revealed metabolic alkalosis, severe hypokalemia, elevated creatine phosphokinase (CPK), and high-sensitive troponin I (hs-troponin I) levels (Table 1). His urine potassium-to-creatinine ratio was 3.7 (mEq/mmol), indicating hypokalemia related to renal potassium wasting. His electrocardiogram (ECG) displayed a sinus rhythm with QT prolongation and a corrected QT interval (QTc) of 703 milliseconds (ms) (Figure 1). His chest X-ray showed unremarkable findings (Figure 2). According to his medication history and clinical presentations, hypokalemia secondary to licorice intoxication-induced pseudo-hyperaldosteronism was under impression.

He was administered a 20 mEq supplement of parenteral potassium chloride. Despite this, the prolonged QT interval persisted, and ventricular premature complexes appeared on his ECG. Follow-up cardiac enzyme tests revealed elevated levels of hs-troponin I at 634.7 ng/L and CPK-MB mass at 7.4 ng/mL. Consequently, he was admitted to the intensive care unit (ICU).

**Figure 3:**
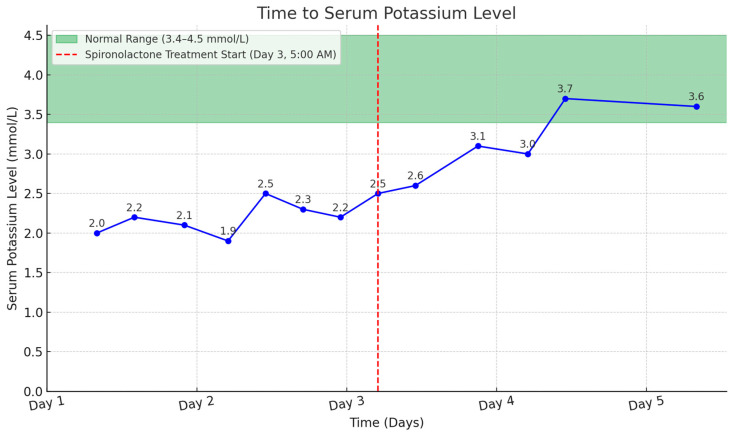
Time to serum potassium level with spironolactone treatment highlighted.

The patient was treated with oral potassium gluconate, receiving 20 mEq four times daily, and intravenous potassium chloride at a dose of 20 mEq in 500 mL of 0.9% normal saline. Additionally, he received an intravenous supplement of 40 mEq potassium chloride in 110 mL of water every six hours. Despite treatment, his hypokalemia did not improve. In response to the persistent hypokalemia, he received spironolactone, an aldosterone receptor antagonist, at a dosage of 25 mg three times daily, beginning on the 3rd day after admission. The introduction of spironolactone, alongside potassium supplements, quickly normalized the patient’s hypokalemia (Figure 3). Furthermore, his metabolic alkalosis and rhabdomyolysis also improved with supportive care.

**Figure 4:**
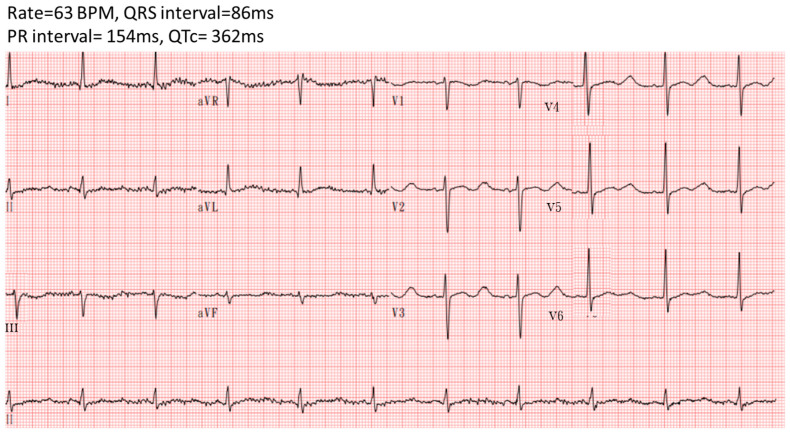
Electrocardiography showed no QTc prolongation after the correction of hypokalemia.

The patient’s condition stabilized, with no further QT interval prolongation or ventricular premature complexes observed with a follow-up ECG after treatment (Figure 4). He was transferred to a general ward on the 4th day and discharged on the 6th day after admission without any complications. He received oral potassium gluconate at 20 mEq four times daily for five days following discharge.

## 3. Discussion

Hypokalemia can result from increased potassium excretion, inadequate dietary intake, the redistribution of potassium into cells, magnesium deficiency, and rare hereditary conditions [3]. A diagnostic evaluation for hypokalemia primarily relies on assessing the urine potassium-to-creatinine ratio and the patient’s acid-base status [4]. Specifically, a spot urine potassium-to-creatinine ratio exceeding 13 mEq/g of creatinine (or 1.5 mEq/mmol) indicates excessive renal potassium excretion [5].

Hyperaldosteronism can be classified as primary, secondary, or pseudo-hyperaldosteronism. Primary hyperaldosteronism involves excessive aldosterone secretion from the adrenal glands, leading to elevated aldosterone and low renin levels. In secondary hyperaldosteronism, the renin–angiotensin system is activated, causing both renin and aldosterone to rise. Pseudo-hyperaldosteronism mimics hyperaldosteronism but with suppressed renin and aldosterone levels [6]. Licorice intoxication is a cause of pseudo-hyperaldosteronism. In this case, serum renin and aldosterone levels were not measured as they were likely influenced by aggressive potassium supplementation in the emergency department [7]. Other causes of pseudo-hyperaldosteronism that induced refractory hypokalemia included excessive cortisol ingestion or adrenal gland tumors. In addition, medication-induced hypokalemia should also be considered in this refractory hypokalemia patient. However, tracing back to the patient’s history, he did not receive exogenous cortisol, diuretic, or other medications that could promote refractory hypokalemia. His clinical presentations, detailed history record, and rapid recovery of hypokalemia after suitable treatment resulted in his licorice intoxication diagnosis. Consequently, he did not receive further image tests for adrenal gland evaluations.

Licorice exerts a mineralocorticoid-like effect through its metabolite, glycyrrhetic acid, by inhibiting the activity of the enzyme type 2 11-β-hydroxysteroid dehydrogenase, which plays a crucial role in converting cortisol into cortisone [8]. As a result, licorice intoxication results in elevated serum cortisol levels. This increase in cortisol activates mineralocorticoid receptors, thereby mimicking the effects of hyperaldosteronism [9]. Prolonged licorice consumption precipitates cortisol-induced mineralocorticoid effects, manifesting as hypoaldosteronism-like symptoms, including reduced renin levels, sodium retention, hypokalemia, elevated blood pressure, and metabolic alkalosis [10]. Excessive ingestion of licorice can lead to hypertension, QT interval prolongation, cardiac arrhythmias, rhabdomyolysis, hypokalemia, elevated CPK levels, metabolic alkalosis, and seizures [11]. 

According to the patient’s medical history, the CK-MB relative index was 0.28% (calculated as 4.7/1636 × 100%). A CK-MB relative index below 3% typically indicates a skeletal muscle source, while an index above 5% suggests a cardiac origin. Based on these data, the elevated cardiac enzyme levels in this patient are more likely attributable to rhabdomyolysis rather than cardiac injury [12]. Since the patient did not have typical chest pain or chest tightness symptoms, he did not receive further cardiac ultrasound or cardiac catheterization after admission.

The management of licorice intoxication includes administering potassium supplements and providing supportive care. The administration of oral and/or intravenous potassium supplements is guided by monitoring serum potassium levels [13]. Gastrointestinal decontamination is generally discouraged in the case of chronic licorice ingestion [14]. Additionally, spironolactone, a competitive aldosterone receptor antagonist, works by opposing aldosterone at the sodium–potassium exchange site within the distal convoluted renal tubule. This promotes the excretion of sodium and water while conserving potassium [15]. Spironolactone serves as a potentially beneficial adjunct in the treatment of licorice intoxication [16]. In our case, the combined use of spironolactone and potassium supplements facilitated the rapid normalization of serum potassium levels.

It is uncommon to encounter cases of licorice-induced toxicity in clinical practice. However, patients with certain psychiatric disorders, such as schizophrenia, may exhibit illogical thinking patterns, which can lead to the chronic use of substances like licorice without an awareness of the associated risks. For clinicians, it is crucial to thoroughly inquire about a patient’s medication and substance use history, particularly in such cases, as licorice toxicity is primarily associated with long-term consumption rather than acute exposure [17].

## 4. Conclusions

The diagnosis of licorice poisoning requires comprehensive approaches, including holistic patient history interviews, physical examinations, and thorough laboratory assessments. This condition induces pseudo-hyperaldosteronism, characterized by hypokalemia, hypertension, and metabolic alkalosis. These symptoms mirror those associated with excessive aldosterone activity yet occur without an actual increase in serum aldosterone levels.

The management of licorice poisoning includes the replenishment of potassium and the use of mineralocorticoid receptor antagonists, such as spironolactone. However, this treatment modality requires further empirical validation to ascertain its effectiveness and safety in cases of licorice toxicity.

## Figures and Tables

**Table 1:** Laboratory data in the emergency department.

Test	Result	Reference Range
**Complete Blood Count**		
WBC count (10^3^/μL)	7.7	3.5–9.1
Hb (g/dL)	13.3	14.0–17.0
Platelet count (×10^3^/μL)	204	157–377
Neutrophil (%)	50.2	39.4–72.6
Lymphocyte (%)	38.1	21–51
**Venous Blood Gas**		
pH	7.449	7.310–7.390
PCO_2_ (mmHg)	46.9	42.0–59.0
PO_2_ (mmHg)	44.2	15.0–50.0
Base Excess (mmol/L)	6.7	−1.5–3.1
HCO_3_ (mmol/L)	31.8	24.0–31.0
O_2_ Saturation (%)	82	
**Serum Biochemistry Examination**		
Creatinine (mg/dL)	1.14	0.7–1.3
Lactate (mmol/L)	3.7	0.5–2.2
Osmolality (mOsm/kg)	288	275–295
CPK (U/L)	1636	30–223
hs-Troponin I (ng/L)	34	≤19.8
CK-MB mass (ng/mL)	4.7	0.6–6.3
Na (mmol/L)	143	136–146
K (mmol/L)	2.0	3.4–4.5
Ca (mg/dL)	8.1	8.6–10.3
Mg (mg/dL)	1.6	1.9–2.7
P (mg/dL)	<1.0	2.5–5.0
**Urine biochemistry examination**		
K(mmol/L)	15.8	-
Cr (mmol/L)	4.26	
Urine K to Cr	3.7	-
Na (mmol/L)	160	-
Osmolality (mOsm/kg)	409	50–1200

WBC, white blood cell; Hb, hemoglobin; CPK, creatine phosphokinase; hs-Troponin I, high-sensitive Troponin I; CK-MB, creatine kinase-MB; and Cr, creatinine.

## Data Availability

The original contributions presented in this study are included in the article. Further inquiries can be directed to the corresponding author.
